# Somatosensory Misalignment: Persistent Referred Sensation After Intercostal‐to‐Musculocutaneous Nerve Transfer

**DOI:** 10.1155/np/3079894

**Published:** 2026-07-20

**Authors:** Ana Carolina Schmaedeke, Fátima Smith Erthal, Claudia Domingues Vargas, Bia Lima Ramalho

**Affiliations:** ^1^ Institute of Biophysics Carlos Chagas Filho, Federal University of Rio de Janeiro, Rio de Janeiro, Brazil, ufrj.br; ^2^ Research, Innovation and Dissemination Center for Neuromathematics, Institute of Mathematics and Statistics, University of São Paulo, São Paulo, Brazil, usp.br

**Keywords:** brain reorganisation, nerve transfer, referred sensation, sensory assessment, traumatic brachial plexus injury

## Abstract

**Introduction:**

Referred sensation (RS) is described as the sensation evoked in the skin areas distinct from the site of stimulation. The study sought to identify and map RS in participants with traumatic brachial plexus injury (TBPI) through a standardised assessment.

**Methods:**

Conducted as an observational study, Experiment 1 involved 19 participants who underwent RS screening by stimulating 22 specific skin areas across both upper limbs, the neck and the face with a cotton swab. In Experiment 2, a comprehensive RS mapping was performed using Semmes–Weinstein monofilaments on the reinnervated forearm of three subjects who exhibited upper limb–chest RS. One participant underwent RS mapping over 6.3 years.

**Results:**

RSs were reported in three of four participants who underwent intercostal‐to‐musculocutaneous nerve (ICN–MCN) transfer and in two participants who underwent ulnar‐to‐musculocutaneous nerve (UN–MCN) transfer. RS within the original donor’s nerve territory (‘right‐way’) was observed following ICN–MCN transfer. Systematic mapping of ‘right‐way’ RS after ICN–MCN transfer revealed a distinctive distribution pattern in the forearm and chest, lacking clear topographical organisation. Remarkably, longitudinal assessment of a single participant revealed a gradual, scattered expansion of RS over time.

**Discussion:**

To our knowledge, this study provides the first systematic mapping of RS in TBPI participants. Persistent ‘right‐way’ RS following ICN–MCN transfer suggested that the significant distance between donor and recipient nerve representations in the primary somatosensory cortex (S1) may limit plastic reorganisation and restrict the disentangling of sensations between the forearm and chest. This insight underscores the complexity of nerve regeneration and opens new avenues for therapeutic approaches focused on sensory function.

## 1. Introduction

Referred sensation (RS) refers to the experience of feeling a sensation in a part of the body other than the area that is being stimulated [1]. While RS has been observed in able‐bodied individuals [2, 3], it is more frequently reported after severe peripheral injuries [4, 5]. Cortical reorganisation and disorganised axonal growth are thought to be key factors contributing to the occurrence of RS after such injuries [6, 7]. When primary sensory inputs are lost, the cortical sensorimotor regions often respond by invading adjacent territories. An example of this is the invasion of face representation into the cortical hand area following hand amputation, potentially due to the unmasking of silent synapses [7–10]. This reorganisation can also be due to increased inputs from intact body parts [11–13]. Alternatively, remaining axons may grow towards targets that differ from those they originally innervated [6, 14, 15].

In addition to amputation, traumatic brachial plexus injury (TBPI) can also lead to loss or decreased sensitivity in the affected limb, often accompanied by pain, RS and phantom sensations [16–19]. The brachial plexus, formed by five nerve roots (C5, C6, C7, C8 and T1), is responsible for the motor, sensory and autonomic innervation of the upper limb [20]. The motor deficit resulting from TBPI can vary depending on the injured roots and may lead to significant functional decline of the upper limb [21].

Current treatments for TBPI include physiotherapy and surgical interventions aimed at reinnervating the segment affected by the injury. A variety of surgical techniques can be employed depending on the extent of the injury, typically with the goal of recovering motor functions [22, 23]. A commonly performed surgical procedure after TBPI is nerve transfer, where an intact donor nerve or fascicle is moved to the injured nerve as distally as possible to reinnervate the affected muscle [21, 24]. In this procedure, special attention is given to isolate the motor component of the donor nerve to enhance motor recovery by reinnervating the recipient muscle [25].

Reports of RS following nerve transfer in TBPI patients have also surfaced [26–29]. When RS is perceived within the donor’s nerve territory, it is described as ‘right‐way’ [4], raising questions about whether the new sensory connections formed through surgery can be integrated into the body map representation via cortical reorganisation. To address this, our study aimed to: (1) identify RS in TBPI participants who underwent various surgical procedures through a standardised assessment and (2) map surgically induced RS in participants who underwent intercostal‐to‐musculocutaneous nerve (ICN–MCN) transfer through longitudinal follow‐up.

## 2. Methods

This observational study involved TBPI participants who were treated at the physical therapy facility of the Institute of Neurology Deolindo Couto, Federal University of Rio de Janeiro (INDC‐UFRJ) and at the National Institute of Traumatology and Orthopedics (INTO). These subjects were clinically evaluated and registered in the laboratory’s digital database on TBPI [30], and data were stored on the Neuroscience Experiment System (NES) platform [31].

Inclusion criteria were age equal to 18 years or older, preserved communication skills and unilateral TBPI. Participants could present different levels of injury, ranging from upper‐trunk lesions to complete injuries (C5–T1). The lesion level was determined by the surgical team through clinical and/ or surgical evaluation, as well as electroneuromyography and magnetic resonance imaging, when available. Importantly, no selection criteria were applied regarding the type of surgical intervention. Therefore, the cohort included patients who had undergone different surgical procedures or no surgical intervention at the time of the evaluation. Exclusion criteria were previous primary or secondary central and/ or peripheral nervous system diseases.

All participants provided written informed consent to participate in the study, in accordance with the Declaration of Helsinki. The study was approved by the ethics committee of INDC‐UFRJ, Brazil (process number: 298.925).

### 2.1. Sensory Evaluation

#### 2.1.1. Experiment 1: RS Screening

**Figure 1:**
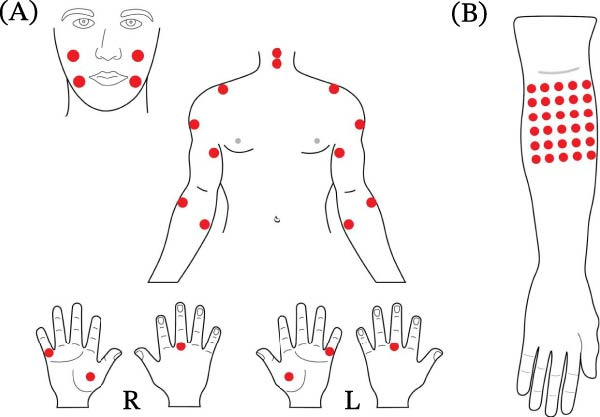
Representation of the stimulated points. (A) Referred sensation screening in Experiment 1. L, left hand; R, right hand. (B) Mapping of the referred sensations in the forearm associated with the intercostal to musculocutaneous nerve transfer in Experiment 2.

Twenty‐two stimulation points distributed between both upper limbs, neck and face were selected for a tactile screening to identify the RS location (Figure 1A). These points corresponded to the dermatomes of the roots from C2 to T2 and to the lower branches of the trigeminal nerve. Stimulation was performed with a cotton swab while the subjects were comfortably seated and blindfolded. The order of stimulation was pseudo‐randomised. Each point was stimulated three times consecutively, and participants were then asked to report if they felt, what they felt (based on a list of sensations but not limited to pricking, burning, squeeze, pressure, vibration, movement, electric shock, touch, itch, heat, cold, tickle, tingling and sweat [free translation from Portuguese]), as well as the location of the sensation, and this information was recorded on a paper sheet. Subjects could point out the site of the sensation with the uninjured upper limb. If the location of the evoked sensation did not correspond to the stimulated point, it was considered RS. If, during the stimuli, no sensation was evoked at a particular point, then it was considered a point of anaesthesia. To avoid inducing the participant to believe that the protocol was specifically designed to evoke their RS, we decided to focus on asking about the sensation type and location (any region of the body) without mentioning that it could be felt in multiple areas. This would avoid any potential conformity bias among participants induced by the experimental protocol [32]. The instructions given before Experiment 1 are given in the Supporting Information. This assessment lasted about 15–20 min.

#### 2.1.2. Experiment 2: Sensorial Mapping of the RS

During the screening performed in Experiment 1, two participants (BPI10 and BPI11) consistently reported evoked sensations in the chest when the lateral forearm was stimulated. These two subjects had undergone ICNs–MCN transfer. To map the chest RS, a new protocol specifically designed for this purpose was applied. The protocol was adapted from that used in amputees to map the sensation of the amputated limb after surgically targeted sensory reinnervation of a pectoral region [33]. Thus, a grid of points was drawn with a dermographic pencil around the autonomous zone of the MCN [34] in the participant’s injured forearm (Figure 1B) to standardise the stimulation area. For stimulation, the filament with the greatest calibre from a set of 20 Semmes–Weinstein monofilaments (Bioseb, Vitrolles, France) was selected to ensure that participants felt the stimuli, avoiding false negatives (based on our previous findings of increased touch thresholds in TBPI participants [19]). Furthermore, with this filament, it was possible to stimulate restricted areas of the skin, allowing detailed mapping of the forearm. This filament exerts a force of 160 g (or 6.20 log of the force [10 × force in mg]) when applied at 90° over the skin, bending into a ‘C’ shape [19]. Participants were asked to sit comfortably, bare‐chested and blindfolded, with the injured arm resting on a pillow. The grid points were then stimulated by applying the filament to each point once. The participants were instructed to say ‘yes’ every time they felt something. Then, the evaluator asked them to report the type of evoked sensation and to point out, with the uninjured upper limb, the region where the sensation was felt. When the RS was reported, its location was delimited with a dermographic pencil. The instructions given before Experiment 2 are in the Supporting Information.

One subject (BPI12), who was not assessed at the RS screening (Experiment 1), was included in Experiment 2. This subject had undergone ICNs–MCN transfer and participated in a touch threshold assessment of both arms in Ramalho et al. [19] 2019 (Figure S2 from Ramalho et al. [19]—participant BPI16). He was the only subject evaluated longitudinally.

## 3. Results

### 3.1. Participants

The study included 20 TBPI participants (three females; mean age: 32 ± 7.21 years, range: 19–44), 19 participating in Experiment 1 and three in Experiment 2. Personal information, including injury level, type of surgery, time between injury and surgery and time between injury and sensory assessment, is presented in Table 1. Nine participants had all brachial plexus roots compromised (C5–T1), and 11 participants had lesions in the upper trunk (C5–C6) or extended upper trunk (C5–C7). One participant had not undergone any surgery at the time of the evaluation, while the other two participants had undergone two surgeries. The interval between the injury and the first surgery ranged from 86 to 464 days (mean: 224 ± 109.20 days), while the interval between the injury and the sensory assessment ranged from 211 to 1396 days (mean: 650.31 ± 311.69 days).

**Table 1:** Subject’s characteristics, description of brachial plexus lesion/surgery.

ID	Gender, age (years)	Handedness	Injury side and extension	Time between injury and surgery	Surgery type	Time between surgery and screening	Referred sensation	Time between surgery and mapping
BPI01	F, 26	Right‐handed	Left, C5–T1	195	SAN–SSN transfer	857	No	—
BPI02	M, 26	Right‐handed	Left, C5–T1	372	Exploration + C5, C6, C7 and SSN neurolysis	81	No	—
BPI03	M, 30	Right‐handed	Right, C5–T1	185	Exploration	284	No	—
BPI04	M, 39	Right‐handed	Left, C5–C7	355	UN–MCN transfer	45	No	—
BPI05	M, 33	Right‐handed	Right, C5–C7	148	SAN–SSN transfer + UN–MCN transfer	63	No	—
BPI06	F, 27	Right‐handed	Right, C5–C7	260	SAN–MCN transfer/UN–MCN transfer	871	No	—
BPI07	M, 24	Right‐handed	Right, C5–C7	—	No surgery	534^a^	No	—
BPI08	M, 32	Right‐handed	Right, C5–C6	177	SAN–SSN transfer + UN–MCN transfer + TRN–AXN transfer	1219	No	—
BPI09	M, 19	Right‐handed	Right, C5–C7	100	UN–MCN transfer	126	No	—
BPI10	M, 31	Ambidextrous	Left, C5–T1	92	SAN–SSN transfer + ICNs–MCN transfer	644	Yes	707
BPI11	M, 38	Ambidextrous	Left, C5–T1	102	ICNs–MCN transfer/SSN neurolysis	798	Yes	833
BPI12	M, 40	Right‐handed	Left, C5–T1	86	SAN–SSN transfer + ICNs–MCN transfer	—	No	79
							Yes	758, 2242
BPI13	M, 29	Right‐handed	Right, C5–C6	324	SAN–SSN transfer + UN–MCN transfer + TRN–AXN transfer	358	Yes	—
BPI14	M, 33	Right‐handed	Left, C5–T1	247	ICNs–MCN transfer	618	No	—
BPI15	M, 44	Right‐handed	Right, C5–C6	344	SAN–SSN transfer + UN–MCN transfer	295	No	—
BPI16	M, 43	Right‐handed	Left, C5–T1	240	SAN–MCN transfer	204	No	—
BPI17	M, 27	Right‐handed	Right, C5–C7	105	UN–MCN transfer	307	Yes	—
BPI18	M, 44	Right‐handed	Left, C5–C6	240	SAN–SSN transfer + UN–MCN transfer	338	No	—
BPI19	F, 25	Right‐handed	Right, C5–T1	229	SAN–SSN transfer	219	No	—
BPI20	M, 29	Right‐handed	Left, C5–C6	464	UN–MCN transfer	316	No	—

*Note:* Time periods are expressed in days. BPI12 participated only in Experiment 2.

Abbreviations: ICNs–MCN, intercostal nerves–to–musculocutaneous nerve; SAN–MCN, suprascapular nerve–to–musculocutaneous nerve; SAN–SSN, spinal accessory nerve–to–suprascapular nerve; TRN–AXN, triceps branch of the radial nerve–to–axillary nerve; UN–MCN, ulnar nerve–to–musculocutaneous nerve.

^a^Time between injury and screening in BPI07.

In participants with a complete brachial plexus injury (C5‐T1), the spinal accessory nerve‐to suprascapular nerve (SAN–SSN) and the ICNs–MCN transfers were the most common surgical interventions. In ICNs–MCN transfer, an attempt was made to separate the sensory components from the motor components of the T3 and T4 nerves that were used as sources of donor nerve for the transfer [35].

In participants with upper trunk lesions, the transfer of a fascicle of the ulnar nerve to the biceps motor branch of the MCN (known as the Oberlin nerve transfer) was the most common.

The participants’ muscle strength on a scale of 0–5 according to the Medical Research Council [36] is shown in Table S1 of the supporting data.

### 3.2. Sensory Evaluation

#### 3.2.1. Experiment 1: RS Screening

**Figure 2:**
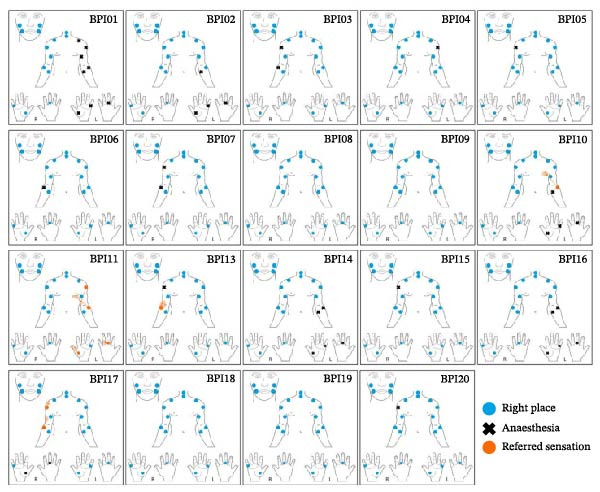
Screening of referred sensations from BPI01 to BPI20. The blue circles represent the points where sensations were evoked in the correct place. Black crosses represent the points of anaesthesia. Orange circles represent the points that evoked referred sensations (indicated by the arrows).

The results of the RS screening are shown in Figure 2. Each picture represents a participant’s sensory deficit profile. Fourteen subjects did not feel any stimulation at one or more stimulation points; these points are hereafter considered points of anaesthesia (black crosses). BPI01 had the worst sensory deficit as none of the stimulated points on the injured arm evoked any sensation, while four subjects had no identified anaesthesia points. Four participants (BPI10, BPI11, BPI13 and BPI17) consistently reported RS (orange circles), whereas the remaining participants did not report any RS. BPI10 and BPI11 reported RS in the chest upon stimulation of the lateral forearm. In contrast, BPI13 exhibited an expanded area of sensation surrounding the stimulated region and BPI17 reported a proximal displacement of the sensation relative to the stimulation site (Figure 2).

#### 3.2.2. Experiment 2: Mapping of the Forearm RS

The participants BPI10, BPI11 and BPI12, who reported RS in the chest when the lateral point of the forearm was stimulated, will be described separately below. All subjects had a motorcycle accident that led to complete left TBPI. Information on participants, type of surgery, time between injury and surgery and time between injury and sensory assessment are shown in Table 1.

##### 3.2.2.1. BPI 10

**Figure 3:**
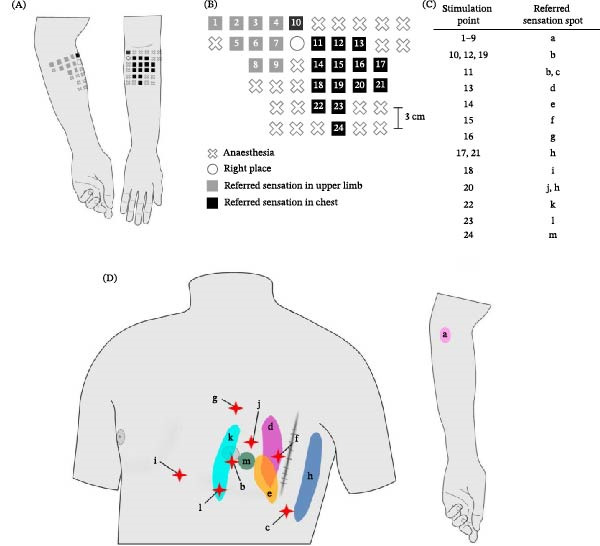
Referred sensation mapping of BPI10. (A) Representation of the stimulated points grid of the forearm. (B) Stimulated points grid—each point is represented according to the participant’s report. (C) Table listing the points that evoked RS on the forearm and the sites of the RS, which can be seen in D. (D) Representation of the RS sites. Red stars represent a pricking sensation.

The sensory mapping of participant BPI10’s forearm can be seen in Figure 3. In total, 46 points were stimulated in the upper limb (Figure 3A), resulting in a grid of sensory responses represented in Figure 3B. From this total, 21 were considered anaesthesia points (indicated by crosses in Figure 3A, B). One point evoked a spatially correct sensation (open circle in Figure 3A, B), nine points evoked RS in the upper limb (grey squares in Figure 3A, B), and 15 points evoked RS in the chest (black squares in Figure 3A, B). These 24 RS points were enumerated, so the precise location of the RS can be seen in Figure 3C. The presence, topological distribution and extent of the RS mapped in the chest and forearm can be appreciated in Figure 3D. RS evoked when stimulating forearm points was spread all over the left chest without any identified type of topographical organisation. Some stimulated points led to unpleasant sensations (pricking sensation—red stars in Figure 3D). The sensations evoked in the forearm (1–9 grey squares in Figure 3B) were always identified in the same spot (‘a’ area on the forearm, Figure 3D).

##### 3.2.2.2. BPI 11

**Figure 4:**
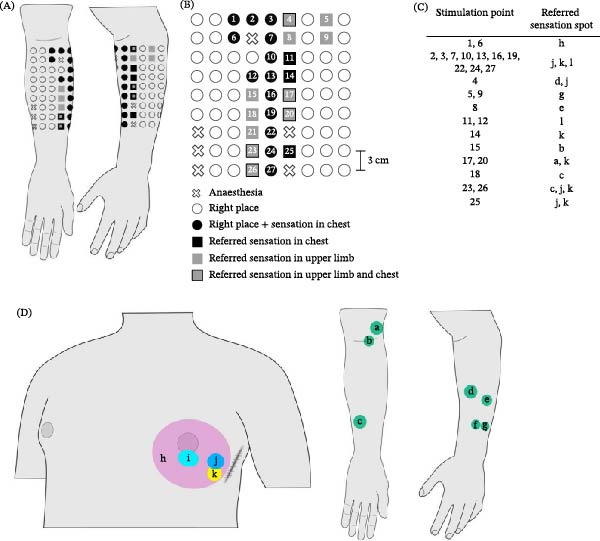
Referred sensation mapping of BPI11. (A) Representation of the stimulated points grid of the forearm. (B) Stimulated point grid—each point is represented according to the participant’s report. (C) Table listing the points that evoked RS on the forearm and the location of the RS, which can be seen in D. (D) Representation of the RS sites.

The sensory mapping of participant BPI11’s forearm is shown in Figure 4. In total, 81 points were stimulated, forming the grid represented in Figure 4A, B. Six were considered as anaesthesia points (crosses in Figure 4A, B) and 48 points evoked correctly placed sensations (open circles in Figure 4A, B). Thirteen points evoked both correctly placed sensations and RS in the chest (black circles in Figure 4A, B), three points evoked RS in the chest (black squares in Figure 4A, B), six points evoked RS in the upper limb (grey squares at Figure 4A, B) and five points evoked RS both in the chest and the upper limb (grey squares with black border at Figure 4A, B). The RS points were enumerated, so the precise location of the RS can be observed in Figure 4C,D.

Unlike BPI10, BPI11 showed a concentration of RS in the chest, while the sensations evoked in the forearm were spread. In this participant, grid points (grey squares with black borders, Figure 4B) in which the stimulation was felt both on the chest and the forearm were observed (Figure 4C, D).

##### 3.2.2.3. BPI 12

**Figure 5:**
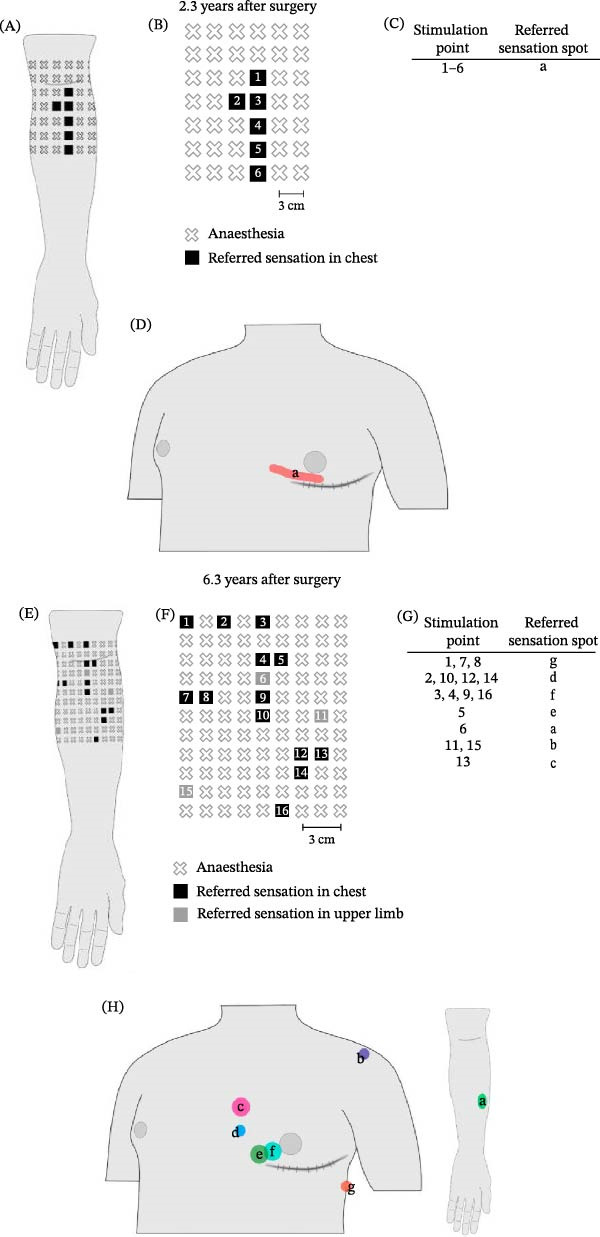
The second and third referred sensation mapping of BPI12. (A) and (E) representation of the stimulated point grid of the forearm. (B) and (F) Stimulated point grid—each point is represented according to the participant’s report. (C) and (G) Table listing the points that evoked RS on the forearm and the sites of the RS, which can be seen in D and H. (D) and (H) Representation of the RS sites.

BPI12 was assessed three times over 6.3 years. The interval between the injury and each assessment was 165, 844 and 2328 days (5.5 months, 2.3 years and 6.3 years, respectively). In its first assessment, BPI12 did not report any RS in the chest when the left forearm was stimulated. Furthermore, the MCN point of exclusive innervation was devoid of any sensation. The mapping performed in the second assessment is shown in Figure 5A–D. The grid had 42 stimulated points, where 36 were considered anaesthesia points (crosses in Figure 5A, B). Six points evoked RS in the chest (black squares in Figure 5A, B), always at the same place (Figure 5C, D).

Finally, mapping was performed again in the third assessment (Figure 5E–H). At this time, from a total of 99 points stimulated, 83 were considered anaesthetised points (crosses at Figure 5E, F), 13 points evoked RS in the chest (black squares at Figure 5E, F) and three points evoked RS in the forearm (grey squares at Figure 5G, H).

Notably, comparing the two mappings reveals a scattering of RS over time. At the first mapping (2.3 years after surgery), stimulated points that evoked RS were close to each other, aligned longitudinally along the forearm, and all of them evoked RS at the same site on the chest. Otherwise, after 4 years, RS was evoked by stimulation of points more distributed across the forearm. Furthermore, the RS evoked at the chest was diffuse, and there was also an RS in the forearm.

## 4. Discussion

In this study, we conducted a systematic and standardised assessment to screen for RS in 20 participants with TBPI. Our goal was to investigate any type of evoked RS in these participants. All participants, except one, underwent surgical treatment, with 17 undergoing at least one of the four types of nerve transfer procedures. Five participants reported RS. In three of the four participants who underwent ICN–MCN transfer, RSs were perceived in the innervation region of the donor’s nerve and were considered “right way”. The systematic mapping of RS in ICN–MCN participants revealed a unique distribution in the forearm and chest, with no clear topographic organisation. A longitudinal assessment of one participant showed that the RS expanded in a scattered manner over time. This report is the first to systematically evaluate this phenomenon in participants with TBPI.

These results are consistent with previous reports that identified RS after ICNs–MCN transfer aimed at motor recovery of the biceps [26–29, 37–39]. One possible explanation for the development of ‘right‐way’ RS after ICNs–MCN transfer is the large number of sensory fibres in the donor nerve [40]. Even with attempts to isolate the motor components of the ICN, some sensory fibres may still be transferred, leading to sensory reinnervation of the forearm and the presence of RS. In contrast, other techniques typically use exclusive motor nerves or motor branches as donor and receptor nerves [41–43], which may limit sensory nerve transfer.

To the best of our knowledge, no RS has ever been reported following an ULN–MCN transfer (Oberlin), a radial‐to‐axillary nerve transfer, or an accessory‐to‐suprascapular nerve transfer. In a study of 78 patients with TBPI, with 54 individuals having undergone nerve transfers, Htut et al. [4] reported ‘right‐way’ RS in 17% of the cohort. Nevertheless, the specific surgical techniques performed in these cases were not explicitly detailed. In our sample, two patients who underwent ULN–MCN transfer (BPI13 and BPI17) also exhibited RSs. In these cases, sensations were elicited in the affected upper limb in areas adjacent to the sites of stimulation. BPI13 presented an expanded area of sensation around the lateral forearm stimulation site. BPI17 reported a displacement of the sensation towards a more proximal region when both the lateral forearm and arm were stimulated. While a potential association between these sensations and the surgical procedures cannot be excluded, their distribution pattern was not compatible with the classical donor nerve territories, although anatomical variations may exist [34, 44].

While nerve transfers in TBPI are primarily aimed at motor recovery—often viewing sensory outcomes as secondary or adverse effects—there has been considerable investigation into sensory reinnervation in peripheral nerve injuries, particularly for restoring protective sensation in the hand [45, 46]. To restore sensitivity in the hand or fingers, the ulnar nerve and the lateral cord of the median nerve are frequently used as primary receptor nerves [46]. Similarly, to restore sensation in the lateral forearm, the sensory branch of the MCN is utilised [47, 48]. Potential sensory nerve donors include the ICN [39, 47, 49–53] and the contralateral C7 root [39, 48, 54]. In many cases, stimulation of the hand or lateral forearm can evoke sensations in the chest as well [38, 49, 55, 56].

In our study, ‘right‐way’ RS evoked by the ICN–MCN transfer was typically scattered, without any sign of topographic distribution in the chest and forearm. Notably, the longitudinal mapping (up to 6.3 years post‐surgery) of RS in one participant (BPI12) indicated a progression from a single RS localised in the chest to a broader distribution with a higher number of RS occurrences. This suggests a lack of directionality in the regeneration of peripheral nerve components. Our findings support previous reports indicating the absence of topographic specificity in sensory nerve regeneration [57–59]. After nerve repair, nerve fibres can grow misdirected, sometimes reaching unintended skin regions. When this occurs, sensation in the reinnervated area can be perceived as coming from the original skin territory of the growing nerve or from the donor’s nerve in the case of nerve transfer [60, 61].

An interesting observation was made in participant BPI10, who was evaluated shortly after surgery and reported experiencing pricking RS. This aligns with the historical data series, as Rivers and Head (1908) (apud [62]) noted that pain and heat sensations are the first to return following a nerve injury and subsequent repair, in their case of the median nerve. These sensations, along with itching and ‘tingling’ (assessed by the Tinel’s test), are primarily carried by unmyelinated fibres, likely c‐fibres, which are typically the first to regenerate [60, 62, 63].

The longitudinal mapping also demonstrated the temporal patterns of RS onset and persistence. RS was absent during the first 5.5 months post‐surgery but was reported after 2.3 years, persisting for a period of 6.3 years. These findings coincide with reports noting RS onset around 12 months following a nerve transfer [27, 28] and even after 6 years post‐surgery [26, 29]. For instance, Nagano et al. [29] found that the majority of 176 participants who underwent the ICNs–MCN transfer experienced the ‘right way’ RS. Interestingly, the sensation in the MCN territory gradually began to manifest to some degree 6 years post‐surgery [29]. The only three cases that exhibited complete switching of sensation from the chest to the upper limb involved participants under 20 years of age, operated on at ages 3, 4 and 8 years, respectively [29]. The persistence of RS following sensory nerve transfer in adult TBPI participants may indicate a unique reorganisation of topographical representations in the primary somatosensory cortex (S1).

Curiously, such reorganisation has been employed in amputees to provide helpful sensory feedback from a prosthetic device [33, 64]. The surgical procedure known as ‘target reinnervation’ consists of transferring residual arm nerves to chest muscles or to the stump to enable the growth of sensory afferents to reinnervate the chest or stump skin. Once reinnervation occurs, tactile stimulation in the reinnervated area evokes a sensation in the missing limb [33, 64]. The persistence of this sensation has been reported in participants up to 16 years after the procedure [64].

A topographic rearrangement in S1 is expected after peripheral injury and nerve transfer [14, 65]. Electrophysiological recording in primate S1 after restricted deafferentation showed an invasion of adjacent representations on the deprived cortex from remnant inputs [66]. However, when the sensory loss is extensive, part of S1 may remain unresponsive [67]. Furthermore, during nerve regeneration, previously well‐defined cortical representations are transformed into scattered, discontinuous representations due to misdirected axonal growth [65]. Chen et al. [54] performed a study with 12 infants with birth injury or TBPI who underwent C7 contralateral root transfer at the age of 6–93 months. During follow‐up (21–63 months post‐surgery), all participants presented synchronous motion and sensibility in the donor’s limb, accompanied by a regain of function in the affected side [54]. Together, these findings support the hypothesis of a constrained S1 reorganisation capacity, potentially explaining persistent RS in longitudinal post‐transfer assessments.

On the motor side, it has been reported that, following an ICNs– MCN transfer, biceps function recovery is associated with topographic reorganisation in the primary motor cortex (M1) [68–70]. Investigating longitudinal changes in M1 of these participants, Mano [68] found that up to 6 months post‐surgery, the biceps representation in M1 was spatially closer to the representation of the trunk. Also, biceps contraction was always associated with respiratory movements [68]. Then, one to 2 years after ICNs– MCN nerve transfer, as participants acquired the ability to flex the elbow in dissociation from respiratory movements, the biceps representation became independent and more lateral than the intercostal representation in M1 [69]. Corroborating these findings, employing a functional magnetic resonance imaging protocol, Malessy et al. [69] showed that, at least 4 years post‐surgery, the centre of gravity of the reinnervated biceps representation appeared more laterally in M1 than that of the intercostal muscle representation. Unfortunately, these studies did not report outcomes related to the sensory component.

## 5. Conclusions

In the present study, three of the four participants with ICNs–MCN transfers exhibited ‘right‐way’ RS, which can be justified by the nerve transfer performed. This phenomenon may reflect constrained S1 plasticity due to the considerable distance between the donor and receptor nerve representations in S1, thereby limiting the disentanglement of forearm and chest sensations. These findings could help clarify the constraints imposed by sensory nerve transfers and aid in the planning of surgical and rehabilitation strategies. This study has limitations, including a small sample size and the need for more detailed surgical data to correlate with RS onset and chest distribution.

## Funding

This work was produced as part of the activities of the Research, Innovation, and Dissemination Center for Neuromathematics of the São Paulo Research Foundation (FAPESP) (Grants #2013/07699‐0 and #2022/00582‐9 to Bia Lima Ramalho) and by the Brain Plasticity after Brachial Plexus Injury projects (Fundação de Amparo à Pesquisa do Estado do Rio de Janeiro (FAPERJ) (Grants CNE #E‐26/202.785/2018, #E‐26/010.002418/2019, CNE #E‐26/204.076/2024, Pensa Rio #260003/020273/2025 and #E‐26/200.349/2025 to Bia Lima Ramalho). It also received funding from FINEP (Proinfra Hospitalar Grant #18.569‐8) and was also supported by the Conselho Nacional de Desenvolvimento Científico e Tecnológico (CNPq) (Grants #310397/2021‐9, #407092/2023‐4 to Claudia Domingues Vargas, #141723/2015‐7 to Bia Lima Ramalho and #150225/2026‐1 to Ana Carolina Schmaedeke). This research was also supported by INCT‐NeuroComp (CNPq Grant #408389/2024‐9) and by INCT NUMEC (CNPq Grant #408590/2024‐6). Ana Carolina Schmaedeke was supported by the Coordenação de Aperfeiçoamento de Pessoal de Nível Superior (CAPES) (Grant #88887.511155/2020‐00).

## Disclosure

A preliminary version of this manuscript was previously published on medRxiv [71] and is available at the following link: https://www.medrxiv.org/content/10.1101/2024.02.19.23297564v1.

## Conflicts of Interest

The authors declare no conflicts of interest.

## Supporting Information

Additional supporting information can be found online in the Supporting Information section.

## Supporting information


**Supporting Information** Document containing the instructions given to each participant before Experiments 1 and 2, and the table showing the participants’ muscle strength on a scale of 0–5, according to the Medical Research Council.

## Data Availability

All relevant data are fully available within the manuscript and in its supporting information files.
