# Supplementary material for: Somatosensory Misalignment: Persistent Referred Sensation After Intercostal‐to‐Musculocutaneous Nerve Transfer
Source: Neural Plast. 2026 Jul 20;2026:3079894. doi: 10.1155/np/3079894 (PMC13384348; doi:10.1155/np/3079894)
Supplement: Supplementary file 1 — Supporting Information Document containing the instructions given to each participant before Experiments 1 and 2, and the table showing the participants’ muscle strength on a scale of 0–5, according to the Medical Research Council. [file NP-2026-3079894-s001.docx]

**Supplementary Method**

**Instructions given before Experiment 1:**

*“This evaluation will be carried out with yourself blindfolded. I will touch some regions of your body with the cotton swab and you must tell me what you are feeling , that means, "how does the sensation feel?" - you must answer with, for example, pricking, burning, squeeze, pressure, vibration, movement, electric shock, touch, itch, heat, cold, tickle, tingling and sweat. After this, you must tell me exactly where you are feeling it (any region of your body). You should be comfortably seated and must remain focused on the assessment at all times.”*

**Instructions given before Experiment 2:**

*"I will start the evaluation by touching a region of the grid on your right (or left) arm with this filament and you should say “Yes” to me each time you feel the touch. Then you must tell me what you felt and point out where you felt it in your body. You can feel it in more than one place at the same time, for example, in two places on your arm or on your arm and chest. After indicating the location of the sensation, I will mark it with the pencil I used to make the dot grid."*

**Supplementary Table 1:** Participants' Muscle Strength According to the Medical Research Council.

| **Table S1 \| Muscular Manual Test** | | | | | | |
| --- | --- | --- | --- | --- | --- | --- |
| **ID** | Shoulder Abduction | Elbow Flexion | Elbow Extension | Wrist Extension | Finger Flexion | Finger Abduction |
| BPI01 | 0 | 0 | 0 | 0 | 0 | 0 |
| BPI02 | 2 | 2 | 1 | 0 | 0 | 0 |
| BPI03 | 0 | 0 | 0 | 3 | 4 | 3 |
| BPI04 | 0 | 2 | 0 | 4 | 4 | 4 |
| BPI05 | 0 | 0 | 4 | 4 | 5 | x |
| BPI06 | 3 | 1 | 3 | 0 | 4 | 0 |
| BPI07 | 2 | 2 | 5 | 5 | 5 | 5 |
| BPI08 | 2 | 5 | 5 | 5 | 5 | 5 |
| BPI09 | 2 | 2 | 5 | 5 | 5 | 5 |
| BPI10 | 0 | 1 | 0 | 0 | 0 | 0 |
| BPI11 | * | 0 | * | 0 | * | * |
| BPI12 2nd | 0 | 0 | 0 | 0 | 0 | 0 |
| BPI12 3rd | 1 | 1 | 0 | 0 | 0 | 0 |
| BPI13 | 1 | 5 | 5 | 5 | 5 | 4 |
| BPI14 | 4 | 0 | 1 | 0 | 0 | 0 |
| BPI15 | 1 | 4 | 5 | 5 | 5 | 4 |
| BPI16 | 1 | 0 | 0 | 0 | 0 | 0 |
| BPI17 | 2 | 3 | 0 | 5 | 5 | 5 |
| BPI18 | 2 | 2 | 3 | 5 | 5 | 4 |
| BPI19 | 0 | 3 | 0 | 0 | 5 | 3 |
| BPI20 | 1 | 3 | 5 | 4 | 5 | 5 |
